# Potential role of pyridostigmine in the management of pediatric chronic intestinal pseudo-obstruction in a girl with ACTL6B mutation: a case report and a review of literature

**DOI:** 10.3389/fped.2026.1761705

**Published:** 2026-04-23

**Authors:** Immacolata Rulli, Angelo Mattia Carcione, Claudio Romano, Roberto Chimenz, Valeria Chirico, Lucia Marseglia, Carmelo Romeo, Eloisa Gitto

**Affiliations:** 1 Neonatal and Pediatric Intensive Care Unit, Department of Human Pathology of Adulthood and Childhood “G. Barresi”, University of Messina, Messina, Italy; 2 Pediatric Gastroenterology and Cystic Fibrosis Unit, Department of Human Pathology of Adulthood and Childhood “G. Barresi”, University of Messina, Messina, Italy; 3 Pediatric Nephrology and Dialysis Unit, Department of Human Pathology of Adult and Childhood “Gaetano Barresi”, University of Messina, Messina, Italy; 4 Pediatric Surgery Unit, Department of Human Pathology of Adult and Childhood “Gaetano Barresi”, University of Messina, Messina, Italy

**Keywords:** ACTL6B mutation, efficacy, PIPO, pyridostigmine, safety

## Abstract

**Background:**

Mutations in the ACTL6B gene are implicated in a wide range of neurodevelopmental disorders, such as global developmental delay, drug-resistant epilepsy, aphasia, autistic traits, dystonia, and cerebral malformations. Recessive variants are typically associated with severe phenotypes, while dominant mutations lead to moderate-to-severe clinical presentations. Although neurological involvement is well characterized, gastrointestinal manifestations, particularly motility disorders, have not been previously associated with ACTL6B-related disease. Pediatric chronic intestinal pseudo-obstruction (PIPO) is a rare, severe gastrointestinal dysmotility disorder. Pyridostigmine, an acetylcholinesterase inhibitor with prokinetic effects, has recently been used off-label in selected PIPO cases.

**Methods:**

We present the clinical course of a 9-year-old female patient with a confirmed pathogenic ACTL6B mutation and a severe neurodevelopmental phenotype. Following persistent feeding intolerance and failure to thrive, the patient underwent surgical intervention, which did not result in significant clinical improvement. Given the suspected neuropathic component of the gastrointestinal dysmotility, pyridostigmine therapy was initiated.

**Results:**

The introduction of pyridostigmine led to improved enteral feeding tolerance, a marked reduction in vomiting episodes, and stabilization of nutritional status, suggesting a potential benefit in managing gastrointestinal symptoms in this patient population.

**Conclusions:**

This case suggests a possible link between ACTL6B-related neurodevelopmental disorders and gastrointestinal dysmotility; if confirmed, it could expand the known clinical spectrum of the disease. Pyridostigmine could be considered as adjunctive therapy in PIPO, especially when a neuropathic etiology is suspected.

## 1 Introduction

### 1.1 Pediatric intestinal pseudo-obstruction (PIPO)

Pediatric Intestinal Pseudo-Obstruction (PIPO), as defined by the European Society for Paediatric Gastroenterology, Hepatology and Nutrition (ESPGHAN) (1), is a rare and severe chronic intestinal motility disorder characterized by ineffective propulsion of gastrointestinal contents in the absence of mechanical obstruction. The condition is considered chronic when symptoms persist for at least two months from birth or for more than six months thereafter. Unlike adult forms, most pediatric cases (up to 80%) are congenital (2) and typically occur sporadically. PIPO results from impaired coordination of the enteric nervous system and/or intestinal smooth muscle, leading to severe dysmotility. Histopathologically, it may be classified as neuropathic, myopathic, or mixed. Secondary forms can be associated with metabolic, mitochondrial, autoimmune, infectious, neoplastic, neuropathic, or drug-induced conditions. Several gene mutations (3), including SOX10 and ACTG2, have been implicated, reflecting the marked genetic heterogeneity of the disorder (4). Diagnosis is based on the exclusion of mechanical obstruction and the presence of objective evidence of neuromuscular dysfunction (5), recurrent bowel dilation, identification of a relevant genetic or metabolic abnormality, and/or failure to maintain adequate growth requiring nutritional support (6). Clinical presentation may begin in the neonatal period and commonly includes abdominal distension, vomiting, constipation, abdominal pain, and feeding intolerance (7). PIPO is associated with significant morbidity and mortality, with complications such as severe malnutrition, recurrent hospitalizations, intestinal failure, and long-term dependence on parenteral nutrition (8). Despite advances in supportive care, outcomes remain poor in a substantial proportion of patients (9). Given its rarity, clinical heterogeneity, and limited therapeutic options, further investigation into its underlying genetic and molecular mechanisms is essential to improve diagnosis, risk stratification, and targeted treatment strategies (10–12).

### 1.2 Pyridostigmine

Among the medications recently explored for use in PIPO, pyridostigmine has gained increasing interest. Pyridostigmine is an acetylcholinesterase inhibitor that prevents the enzymatic breakdown of acetylcholine at neuromuscular junctions, thereby enhancing cholinergic transmission and improving muscular contractility and peristalsis (13). It is available in both oral (tablets, syrup) and parenteral (intravenous) formulations. The efficacy of pyridostigmine is dose-dependent and its dosage must be carefully titrated based on the patient's clinical response and tolerability. The most common adverse effects are cholinergic in nature, including abdominal cramps, diarrhea, nausea, hypersalivation, bronchial hypersecretion, muscle fasciculations. Contraindications include mechanical gastrointestinal or urinary tract obstruction. Caution is advised in patients with asthma due to the risk of bronchoconstriction (14).

### 1.3 ACTL6B and related disorders

ACTL6B (Actin-like 6B) gene plays a critical role in neuronal development. It encodes a component of the neuron-specific BAF (nBAF) chromatin remodeling complex, which is essential during neurogenesis. As neural progenitor cells mature into post-mitotic neurons, the composition of the chromatin remodeling complex changes. Specifically, ACTL6A is replaced by ACTL6B. This subunit exchange is crucial for the recruitment of the nBAF complex and the CREST coactivator, which together regulate neuronal maturation and dendritic arborization (15, 16). ACTL6B is predominantly expressed in the brain and pituitary gland, and its sub-cellular localization includes the nucleolus and mitotic chromosomes (17). The acronym “DECAM syndrome” has been proposed to summarize the core clinical features: Developmental delay, Epileptic encephalopathy, Cerebral atrophy and Abnormal Myelination (18). Pathogenic variants in ACTL6B are associated with a spectrum of neurodevelopmental disorders. Biallelic (recessive) mutations are associated with more severe phenotypes and profound neurological impairment, whereas heterozygous “*de novo*” variants (dominant inheritance) lead to moderate-to-severe phenotypes with intellectual disability, often without seizures or motor involvement. Homozygous “loss-of-function” variants in ACTL6B have also been linked to autism spectrum disorder, suggesting a role in social-cognitive development (19, 20). To date, no specific gastrointestinal manifestations have been reported in ACTL6B-related disorders.

## 2 Case description

We report the case of a 9-year-old girl with a complex neurodevelopmental disorder characterized by severe spastic tetraparesis, intellectual disability and drug-resistant epilepsy, associated with a pathogenic variant in the ACTL6B gene. Nutritional support had been provided via percutaneous endoscopic gastrostomy (PEG), and the patient had previously undergone Nissen fundoplication (Table 1). The patient was admitted to the Pediatric Intensive Care Unit for acute respiratory failure, fever, and recurrent vomiting. Chest radiography revealed right upper lobe consolidation, suggestive of aspiration pneumonia. Her parents reported multiple episodes of vomiting and suspected aspiration events over the preceding three months. During the first days of hospitalization, the patient showed poor tolerance to enteral nutrition via PEG, with frequent vomiting episodes. Due to persistent enteral intolerance, parenteral nutrition (PN) was initiated to meet her caloric and metabolic needs. To exclude anatomical causes of intestinal obstruction, a gastrointestinal transit study was performed, demonstrating regular passage of contrast through the stomach and jejunoileal loops, with delayed gastric emptying. Pharmacologic treatment with ondansetron and prucalopride was ineffective. The PEG-J tube was removed, and the patient subsequently underwent surgical intervention with esophagogastric disconnection, Roux-en-Y reconstruction, and gastrostomy revision. After surgery, gradual reintroduction of enteral nutrition was attempted, starting with a rehydration solution followed by a hydrolyzed formula; however, this was unsuccessful due to repeated regurgitation and gagging. Intestinal transit imaging demonstrated slow progression of contrast to the ascending colon. Abdominal CT excluded mechanical obstruction. Given the persistence of vomiting, the patient underwent exploratory laparotomy with ileostomy placement. Following multidisciplinary evaluation, a clinical diagnosis of pediatric chronic intestinal pseudo-obstruction (PIPO) was suspected. Therapy with pyridostigmine at 0.25 mg/kg/day was initiated in combination with total parenteral nutrition. The patient showed gradual clinical improvement, with reduced vomiting and improved tolerance of minimal enteral feeding. Enteral nutrition was progressively reintroduced using an amino acid–based formula combined with partial parenteral nutrition. The pyridostigmine dosage was gradually reduced to 0.15 mg/kg/day. After several months of hospitalization, the patient was discharged in good general condition, with instructions to continue therapy.

**Table 1:** Timeline of interventions.

Time	Interventions
Admission	Admission for acute respiratory failure, fever and vomiting
First week from admission	Exclusion of post-viral gastroparesis and mechanical obstruction
Second week from admission	Treatment with prucalopride and ondansetron ineffective
Third week from admission	Failed attempts of EN and PEG-j positioning
One month from admission	Nutritional surgery (esophagogastric disconnection, Roux-en-sac reconstruction and gastrostomy revision
2 weeks post-operatively	Persistence of feeding intolerance: ileostomy created
4 weeks post-operatively	After multidisciplinary briefing, pyridostigmine 0.25 mg/kg/day was started
10 days after therapy start	Reduction to 0.15 mg/kg/day after improvement of symptoms
3 months from admission	Discharge

## 3 Discussion

### 3.1 Review of literature

Management of Chronic Intestinal Pseudo-Obstruction (PIPO) remains largely supportive, with a focus on nutritional therapy, symptom control, and prevention of complications. Although pyridostigmine is an established treatment for myasthenia gravis, its use in gastrointestinal motility disorders has only recently been explored. As an acetylcholinesterase inhibitor, pyridostigmine enhances cholinergic signaling within the enteric nervous system, thereby promoting intestinal peristalsis. This mechanism offers a pathophysiological rationale for its potential role in patients with suspected or confirmed neuromuscular dysfunction (21). In the case presented, the patient showed improvement in gastrointestinal symptoms following the introduction of pyridostigmine. This clinical response is aligned with the limited literature supporting the off-label use of pyridostigmine in PIPO (Table 2). To date, randomized controlled trials (RCTs) in children with PIPO are lacking. However, studies in adults have demonstrated that pyridostigmine can enhance intestinal transit and improve stool consistency in patients with autonomic neuropathy, diabetes-related dysmotility, and severe constipation (22–24). Similar benefits have been observed in patients with systemic sclerosis (25) and post-operative ileus (26, 27). In pediatrics, evidence is limited to case reports and small case series. One case report described a child with PIPO who experienced significant clinical improvement after initiating pyridostigmine therapy (28). In another case, a 2-year-old girl receiving 2–3 mg/kg twice daily showed reduced abdominal distension, decreased dependence on parenteral nutrition and improved oral intake, without significant side effects (29). A small case series of children with ACTG2 mutations reported symptomatic improvement and enhanced colonic motility in 2 out of 3 treated patients (30). A 2018 case series demonstrated that oral pyridostigmine had good efficacy and safety in children with various types of gastrointestinal motility disorders (31). A 2022 systematic review emphasized pyridostigmine as a promising agent in PIPO management. Similarly, a 2021 scoping review concluded that acetylcholinesterase inhibitors, including pyridostigmine and neostigmine, were well tolerated and effective in improving motility in children. More recently, a 2023 adult-focused systematic review reported improved outcomes and a low incidence of adverse effects (4.3%), which were generally mild (32). Despite these promising findings, evidence remains limited due to small sample sizes, heterogeneous populations, and the lack of controlled clinical trials in children. Pyridostigmine remains an off-label treatment and should be administered under specialist supervision. Clinical experience suggests a more favorable response in patients with a neuropathic etiology compared to myopathic subtypes, but the lack of validated scales complicates objective assessment of therapeutic efficacy.

**Table 2:** Main evidences about pyridostigmine use in PIPO.

Title	Authors and year	Study type	Patients	Dosage	Main results
*Application of Pyridostigmine in Pediatric Gastrointestinal Motility Disorders: A Case Series*	Manini ML et al., 2018 (31)	Case series	Various GI disorders incl. PIPO; children to adolescents	0.25–2 mg/kg/day	Reduced distension, increased bowel movements, improved enteral feeding, good tolerance
*Pyridostigmine in Pediatric Intestinal Pseudo-obstruction: Case Report of a 2-year Old Girl and Literature Review*	Di Nardo G et al., 2019 (29)	Case report + literature review	2 y; review of prior cases	2 mg/kg BID → 3 mg/kg BID	Reduced distension, resolved vomiting, spontaneous bowel movements
*The Use of Pyridostigmine in a Child With Chronic Intestinal Pseudo-Obstruction*	Choudhury A et al., 2018 (28)	Case report	9 y, PIPO with myopathy	0.5 mg/kg BID → 1 mg/kg BID	Reduced distension, better enteral flow, no significant adverse effects
*Pyridostigmine as a therapeutic option for pediatric gastrointestinal dysmotilities in ATR-X syndrome. Case report and literature review*	Comisi FF et al., 2024 (33)	Case report (ATR-X syndrome)	10 y	1.6 mg/kg/day → 3.2 mg/kg/day	Resolved GI symptoms, better quality of life, no significant side effects
*Pyridostigmine in chronic intestinal pseudo-obstruction—a systematic review*	Wilkie et al., 2023 (32)	Systematic review	Mostly adults, includes some adolescents	Various	Improved symptoms and transit, minimal side effects (<5%)
*Oral Pyridostigmine-responsive Visceral Myopathy With ACTG2 Mutations: A Case Series*	Lee H et al., 2019 (30)	Case series	Children with ACTG2 mutations (visceral myopathy)	Not specified (oral pyridostigmine)	Clinical improvement in motility symptoms, good tolerance
*Pediatric Intestinal Pseudo-Obstruction: Progress and Challenges*	Turcotte MC et al., 2022 (8)	Review	N/A	N/A	Comprehensive review of etiology, diagnosis, treatment and future directions
*Paediatric intestinal pseudo-obstruction: a scoping review*	Nham S, et al., 2022 (2)	Scoping review	N/A	N/A	Broad literature synthesis on pediatric PIPO epidemiology, management and outcomes

### 3.2 Limitations of the study

Our report has several important limitations that should be carefully acknowledged. This observed combination represents a preliminary clinical hypothesis and not a demonstrated causal relationship. Indeed, it is limited by its single-case design, which does not allow any unmistakable link between ACTL6B related diseases and pediatric intestinal pseudo-obstruction (PIPO). In a child with a complex neurodevelopmental disorder, gastrointestinal dysmotility could be multifactorial. Alternative explanations, including secondary dysmotility, incidental comorbidity, or additional undetected genetic variants cannot be excluded. Therefore, the proposed genotype–phenotype association remains speculative. Moreover, no functional or mechanistic studies were performed to support a direct role of ACTL6B in enteric neuromuscular dysfunction, neither a functional evaluation (e.g., gastrointestinal manometry) or a direct pathological testing (e.g biopsy of the enteric plexus) were conducted in this patient. In the absence of *in vitro* or *in vivo* data, the hypothesized link must be interpreted cautiously. Finally, the observed clinical improvement with pyridostigmine is based just on descriptive observation without standardized outcome measures; no data about long-term follow-up were available to address conclusions regarding efficacy, optimal dose and safety. Both the proposed pathogenic association and the therapeutic implications should be thus interpreted with caution.

### 3.3 Future directions

Further studies are needed to clarify if a link really exists between ACTL6B-related disorders and gastrointestinal dysmotility. Collection of additional well-characterized cases through multicenter collaborations or registry-based studies could help. Functional investigations exploring the role of ACTL6B in enteric nervous system development or smooth muscle regulation would be particularly valuable to assess biological plausibility. Experimental models could provide insight into potential mechanisms underlying gastrointestinal involvement. Finally, prospective studies assessing pyridostigmine in pediatric patients with neuromuscular-related dysmotility, using standardized outcome measures and extended follow-up, are required to better identify responsive subgroups, establish optimal dosing regimens, and evaluate long-term safety.

## 4 Conclusions

ACTL6B-related disease is primarily characterized by neurodevelopmental impairment; however, this case suggests a possible association with gastrointestinal involvement, including pediatric intestinal pseudo-obstruction (PIPO). Although this observation does not establish a causal relationship, it highlights the need for further investigation into potential gastrointestinal manifestations in this genetic condition. Pyridostigmine may represent a promising adjunctive therapeutic option in selected pediatric patients with PIPO and underlying neuromuscular or genetic disorders, potentially improving symptoms such as abdominal distension, feeding intolerance, and nutritional status. Nevertheless, current evidence remains limited, and its efficacy and safety in this specific context require further confirmation. Therapeutic management should be individualized and guided by a multidisciplinary team. Future studies are needed to clarify the potential link between ACTL6B-related disease and gastrointestinal dysfunction, to identify patients who may benefit from pyridostigmine therapy, and to define optimal dosing strategies and long-term outcomes.

## Data Availability

The original contributions presented in the study are included in the article/Supplementary Material, further inquiries can be directed to the corresponding author/s.
