# Correction: Potential role of pyridostigmine in the management of pediatric chronic intestinal pseudo-obstruction in a girl with ACTL6B mutation: a case report and a review of literature

**DOI:** 10.3389/fped.2026.1958105

**Published:** 2026-08-28

**Authors:** Immacolata Rulli, Angelo Mattia Carcione, Claudio Romano, Roberto Chimenz, Valeria Chirico, Lucia Marseglia, Carmelo Romeo, Eloisa Gitto

**Affiliations:** 1 Neonatal and Pediatric Intensive Care Unit, Department of Human Pathology of Adulthood and Childhood “G. Barresi”, University of Messina, Messina, Italy; 2 Pediatric Gastroenterology and Cystic Fibrosis Unit, Department of Human Pathology of Adulthood and Childhood “G. Barresi”, University of Messina, Messina, Italy; 3 Pediatric Nephrology and Dialysis Unit, Department of Human Pathology of Adult and Childhood “Gaetano Barresi”, University of Messina, Messina, Italy; 4 Pediatric Surgery Unit, Department of Human Pathology of Adult and Childhood “Gaetano Barresi”, University of Messina, Messina, Italy

**Keywords:** ACTL6B mutation, efficacy, PIPO, pyridostigmine, safety

Boxes

Wrong citation

In the published article, there was a mistake in the Table 2 “Main evidences about pyridostigmine use in PIPO”. In the fourth box, “Pyridostigmine as a therapeutic option for pediatric GI dysmotilities in ATR-X syndrome” was incorrectly cited as “Boybeyi Türer et al., 2020 (7)”. The correct citation is:

Article title: Pyridostigmine as a therapeutic option for pediatric gastrointestinal dysmotilities in ATR-X syndrome. Case report and literature review”

Authors and year: “Comisi FF et al., 2024 (33)’’.

The full citation should also be listed in References as “33. Comisi FF, Soddu C, Corpino M, Marica M, Cacace R, Foiadelli T, Savasta S. Pyridostigmine as a therapeutic option for pediatric gastrointestinal dysmotilities in ATR-X syndrome. Case report and literature review. Front Pediatr. 2024 Dec 17;12:1460658. doi: 10.3389/fped.2024.1460658. PMID: 39741769; PMCID: PMC11685138.”

The original version of this article has been updated.

